# Recurrent Chylous Pleural Effusions in a Patient Treated With Dasatinib

**DOI:** 10.7759/cureus.27778

**Published:** 2022-08-08

**Authors:** Syed Alishan Nasir, Hugo Calavia Liano, Christopher Manfredi

**Affiliations:** 1 Internal Medicine, Norwalk Hospital, Norwalk, USA; 2 Pulmonary and Critical Care Medicine, Norwalk Hospital, Norwalk, USA

**Keywords:** drug-related side effects and adverse reactions, dasatinib, thoracentesis, exudative pleural effusion, rare cause of pleural effusion, chylous effusion, chronic myeloid leukemia (cml), imatinib mesylate, tyrosine kinase receptor inhibitors

## Abstract

Dasatinib is a second-generation BCR-ABL tyrosine kinase inhibitor (TKI) that is approved for the treatment of chronic myeloid leukemia (CML), a myeloproliferative disorder seen commonly in adults over the age of 50. Dasatinib is superior in tolerance and efficacy to first-generation TKIs such as imatinib, given its ability to target mutation products that are resistant to first-generation TKIs. One of the common side effects of dasatinib includes pleural effusion which can be seen in up to 25% of patients on treatment. These effusions are predominantly exudative; however, they tend to resolve upon discontinuation of the drug. While infrequent, chylous effusions have been reported with the use of dasatinib; they tend to resolve following discontinuation of the drug. We present a case of a patient who was treated with dasatinib and developed a chylous effusion which was refractory to the discontinuation of the medication. Our patient was switched to imatinib and since his first episode, he has had multiple reaccumulation requiring thoracentesis, all of which have revealed chylous pleural fluid as per fluid analysis. We present this case to highlight a rare adverse effect of dasatinib which, via an unknown mechanism, can potentially lead to irreversible damage to the lymphatic duct resulting in recurrent chylous effusions.

## Introduction

Dasatinib is an orally active small molecule inhibitor of multiple tyrosine kinases including BCR-ABL and SRC family kinases and is indicated for the treatment of adults with newly diagnosed chronic myeloid leukemia (CML), particularly in patients with resistance or intolerance to prior therapy with imatinib [1-2]. Amongst the non-hematological side effects reported with dasatinib, the most common ones include fluid retention, diarrhea, headache, nausea, skin rash, dyspnea, hemorrhage, fatigue, musculoskeletal pain, infection, vomiting, cough, abdominal pain, and pyrexia [2]. Pleural effusions comprise the major component of fluid retention adverse events which also include superficial edema and rarely pericardial effusions. In most cases, these pleural effusions are exudative and are normally, successfully manageable with dasatinib dose interruption and/or concomitant diuretic or corticosteroid use [2-3]. Despite pleural effusions being a common side effect associated with dasatinib use, a chylous effusion is rarely seen. Additionally, this too has been shown to resolve with discontinuation of the drug; however, in our case, the patient continues to have these recurrent chylous effusions despite discontinuation of dasatinib.

## Case presentation

A 74-year-old male with a past medical history of CML in molecular remission, on treatment with low dose dasatinib 80mg daily, presented to the oncologist’s office in August 2018 for worsening dyspnea. A chest X-ray (CXR) was obtained which showed a moderate to large right-sided pleural effusion. At this time, dasatinib was discontinued, and the patient was then referred to our pulmonology office; he underwent a thoracentesis with the removal of 1.3L of pink, milky-appearing fluid grossly consistent with a chylous effusion. Fluid analysis showed an exudative fluid with pleural fluid total protein/serum total protein ratio > 0.5. Pleural fluid triglyceride level was 1,123.mg/dL (triglyceride level > 110mg/dL is diagnostic for chylous effusion). Cytology of pleural effusion was negative for malignant cells. The patient initially experienced an improvement in symptoms and a CXR confirmed resolution, however, his dyspnea returned about four weeks later and a CXR at this time showed an interval increase in pleural effusion prompting a second thoracentesis with the removal of 0.8L of opaque, pink, milky appearing fluid which was also grossly consistent with a chylous effusion. No fluid analysis was obtained at this time.

The patient was then started on imatinib therapy 400mg daily. Two months later, the patient developed dyspnea again and a CXR showed a right-sided pleural effusion. A third thoracentesis was then performed and fluid studies were consistent with an exudative effusion, a triglyceride level of 492mg/dL, and pleural fluid cholesterol level of 46mg/dL, indicating a chylous effusion. A repeat CXR showed resolution of the effusion. The patient then underwent a fourth thoracentesis with the removal of 1.3L on December 20, 2018 which was also negative for malignant cells on cytology. A post-procedure CXR showed interval resolution of right-sided pleural effusion.

The patient was then followed at the pulmonology clinic and was noted to have reaccumulating pleural effusion in late January of 2019 which was seen on CXR. He then underwent his fifth thoracentesis with the removal of 1.1L of chylous effusion. He remained asymptomatic for a while, however, he subsequently developed right-sided pleuritic chest pain around July 2019 for which he was evaluated in the ED. A computed tomographic angiogram (CTA) of the chest showed a large right-sided pleural effusion and he underwent repeat thoracentesis with the removal of 0.9L of pink, milky fluid. Fluid analysis at this time also demonstrated an exudative effusion with a pleural TG level of 648mg/dL and a pleural cholesterol level of 113mg/dL. Post-procedural CXR showed resolution of effusion.

**Figure 1:**
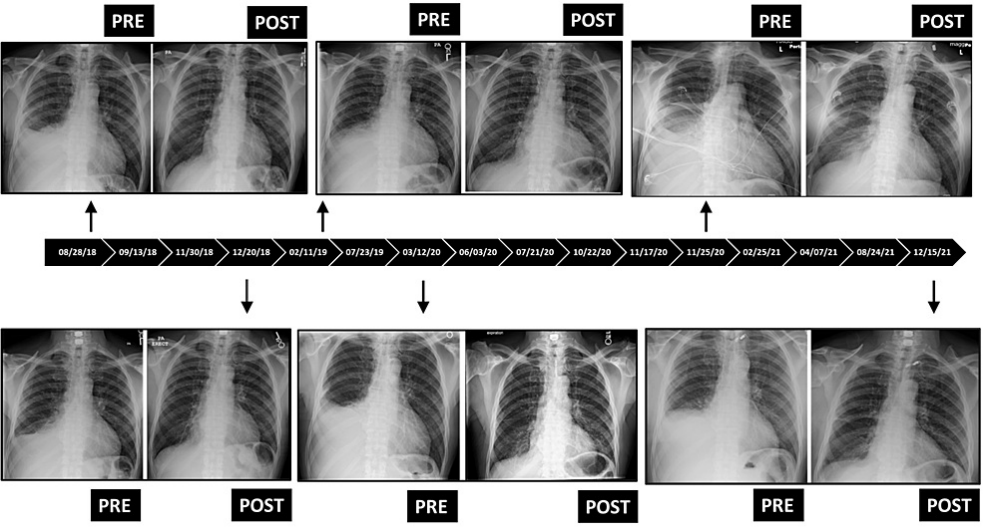
Sample chest X-rays of the patient pre- and post-thoracentesis over a four-year span highlighting the resolution of the right-sided pleural effusion. Timeline reveals the dates when each thoracentesis was performed.

Since July 2019, the patient has had multiple recurrences of right-sided pleural effusions requiring multiple thoracentesis. He was monitored for expansion of pleural effusion with serial CXRs and underwent a total of six thoracentesis in 2020, all of which were opaque and milky (chylous) in appearance. Pathology was persistently negative for carcinoma or malignant cells. An MRI of the chest was performed to look for anatomical defects in the lymphatic duct, however, the lymphatic duct could not be visualized and hence the study was non diagnostic. He had a similar clinical course in 2021 as well. His imatinib was discontinued for three months, however, he was noted to have a relapse of CML and hence, imatinib was resumed. He was referred for a lymphangiogram at a tertiary care center which did not find any obvious defects but a coil was placed in the lymphatic duct. Despite this, he continued to have recurrent chylous effusions on the right side. A timeline with the patient’s procedures and CXR findings is shown in Figure 1. The patient is currently being followed at our pulmonology office and continues to have recurrent chylothorax. A steroid trial was not successful. He is currently on imatinib and is tolerating the medication otherwise. His CML is also in relapse now and he is regularly monitored for reaccumulating pleural effusion via serial CXRs and undergoes thoracentesis as needed. Table 1 shows the dates during which pleural fluid analysis was obtained and the triglyceride level and cholesterol level noted in the pleural fluid. Note that due to the recurrence of chylous effusions, pleural fluid analysis was not performed each time. 

**Table 1:** Dates corresponding to pleural fluid analysis; columns show the triglyceride and cholesterol levels observed in the pleural fluid.

Date of thoracentesis	Pleural fluid triglyceride level (normal < 110mg/dL)	Pleural fluid cholesterol level
08/28/2018	1,123 mg/dL	NA
11/30/2018	492 mg/dL	46 mg/dL
07/23/2019	648 mg/dL	113 mg/dL
01/17/2020	626 mg/dL	110 mg/dL
06/03/2020	1,220 mg/dL	78 mg/dL

## Discussion

Multiple studies have documented pleural effusions to be a common side effect of dasatinib use and these have been seen in 14%-32% of patients that are on treatment with dasatinib [3]. Some risk factors are more closely associated with the development of pleural effusions with dasatinib, and these include prior history of cardiac disease, hypertension, twice daily dosing schedule, and older age [3-5]. Chylous effusions are however rare occurrences and to our knowledge, less than 10 case reports have been published that highlight this as a potential side effect of dasatinib. Chylothorax, by definition, is the presence of chyle in the pleural space. Chylothorax results from any disruption to the thoracic duct. It can be classified as traumatic or non-traumatic. The leading causes of chylothorax are thoracic surgery and malignancy, respectively. Medications typically do not cause chylous effusions. On literature review, dasatinib is one of the few medications associated with the development of chylous effusion.

There have been multiple case reports that document the relationship between dasatinib use and the development of chylous effusion. In one case report, a 40-year-old female with CML, on dasatinib, was found to have developed a chylous effusion. Under the suspicion of drug-related chylothorax, dasatinib was discontinued. Diuretics and steroids were prescribed for symptom control. Her pleural effusion improved after nine days of treatment. Dasatinib was resumed two weeks after discontinuation and the pleural effusion recurred soon while under treatment with diuretics and steroids. Due to intractable chylothorax even after repeated thoracentesis, dasatinib was discontinued again and the patient was later switched to nilotinib. The pleural effusion resolved gradually. After several months of nilotinib use, no further symptoms of pleural effusion were experienced [4].

In the case report by Chen et al., [6] the authors noted resolution of chylothorax after one week of dasatinib discontinuation which was confirmed by ultrasound (as an absence of an anechoic region in the thoracic cavity) as well as improvement in the patient’s dyspnea. The authors then resumed dasatinib for two days with close monitoring and noted the formation of a 3.1 cm anechoic zone in the right thoracic cavity. With this finding, the authors were able to confirm the diagnosis of dasatinib-induced chylothorax. This case report highlighted the linear association of dasatinib use and the successive development of a chylous effusion which immediately resolves once the drug is discontinued. In our patient, however, the chylous effusions appeared to persist despite being off dasatinib for a prolonged period, hence raising the possibility of whether dasatinib possesses a permanent pathological mechanism that gives rise to recurrent chylous effusions. Alternatively, however, it is also possible that chylous effusions may be a rare complication of the tyrosine kinase inhibitor (TKI) family. During our literature review, we could not find any case reports that discussed the development of a chylous effusion from any other TKIs.

In another case report, the authors demonstrated a patient who had recurrent chylothorax while on dasatinib requiring multiple thoracentesis, however, changing the medication to nilotinib followed by an outpatient follow-up for one year revealed no recurrence [7]. Similarly, another report described a 69-year-old male who developed chylothorax after 10 months of treatment with dasatinib following failure with imatinib and nilotinib. He was initially treated symptomatically for the effusion however it soon returned, prompting the discontinuation of dasatinib and switching to bosutinib after which the patient tolerated the therapy well with no recurrence of the chylothorax [8].

The mechanism by which dasatinib causes pleural effusions has not been fully studied, however, it is hypothesized that it may cause chylothorax due to inhibition of platelet-derived growth factor receptor beta or the SRC family kinases. SRC is a proto-oncogene encoding a non-receptor tyrosine kinase which is widely expressed in hematopoietic cells in lung tissue. It is responsible for capillary integrity as it induces the expression of vascular endothelial growth factors. Inhibition of SRC kinases results in changes in vascular permeability as well as pleural epithelium, either of which could eventually affect lymphatic drainage, resulting in chylous effusions [7]. Another possible mechanism of the development of this complication could be that dasatinib inhibits tyrosine kinase platelet-derived growth factor receptor beta (PDGFR-β), which regulates angiogenesis, lymphangiogenesis, and vascular smooth muscle cell proliferation. This in turn results in microangiopathy and defective vascular remodeling that can then lead to leakage of lymph fluid into the pleural space [6-9].

To date, there has been only one case report to our knowledge that has shown a patient with dasatinib-induced chylothorax refractory to discontinuation of dasatinib and switching to a different TKI [9]. Our findings are consistent with the findings of Sasaki et al. who published a case report on dasatinib-induced chylothorax in 2019 where a 79-year-old female with CML, on dasatinib for one year, developed recurrent chylothorax that did not respond to treatment change to bosutinib [9]. This is the only case report that we were able to find in our literature review which documented a case that had a similar presentation as our patient with recurrent dasatinib-induced chylothorax, despite being switched over to a different TKI. During our literature review, no article has surfaced that reports chylothorax associated with other TKIs (imatinib, nilotinib bosutinib).

## Conclusions

Chylous effusions are not commonly seen in clinical practice and setting aside malignancy and tuberculosis, there are only a handful of etiologies. Based on our literature review, there have been consistent case reports discussing this adverse effect of dasatinib. In most cases, however, the chylous effusions appeared to have resolved following discontinuation of dasatinib, however, this was not the case with our patient. We have also failed to find alternative etiologies to our patient's presentation. Additionally, our patient continues to do well with imatinib therapy and over four years, his pulmonary symptoms have been managed effectively with the serial CXRs and thoracentesis. We present this case to highlight an interesting and unusual complication associated with dasatinib use and hypothesize that there may be a class effect associated with TKI which can potentially lead to irreversible lymphatic duct damage causing recurrent chylous effusions. Additionally, another hypothesis may be that certain patients may have a genetic component that sensitizes them to this complication of dasatinib/TKI use. It is for this reason that further investigation into the mechanism by which dasatinib gives rise to chylous effusions may be required.
